# Resveratrol Has Histone 4 and Beta-Defensin 1-Mediated Favorable Biotherapeutic Effects on Liver and Other Target Organs in Diabetic Rats

**DOI:** 10.5152/tjg.2024.23068

**Published:** 2024-03-01

**Authors:** Alpaslan Tanoğlu, Fatih Özçelik, Fatih Hacımustafaoğlu, Gülfidan Coşkun, Tansel Sapmaz, Esra Güzel Tanoğlu

**Affiliations:** 1 Department of Gastroenterology, Bahçeşehir University Faculty of Medicine, Göztepe Medical Park Hospital, İstanbul, Turkey; 2 Department of Medical Biochemistry, University of Health Sciences, Şişli Etfal Training and Research Hospital, İstanbul, Turkey; 3 Department of Medical Biochemistry, University of Health Sciences Hamidiye Faculty of Medicine, İstanbul, Turkey; 4 Department of Histology and Embryology, Çukurova University Faculty of Medicine, Adana, Turkey; 5 Department of Histology and Embryology, University of Health Sciences Hamidiye Faculty of Medicine, İstanbul, Turkey; 6 Department of Molecular Biology and Genetics, Institution of Hamidiye Health Sciences, University of Health Sciences, İstanbul, Turkey

**Keywords:** Diabetes, histone 4, liver, rat, resveratrol

## Abstract

**Background/Aims:**

: It was aimed to investigate the biochemical and histopathological effects of resveratrol and melatonin, via histone H4 and β-defensin 1, in diabetic rats.

**Materials and Methods:**

Twenty-four Sprague–Dawley male rats were categorized into 4 groups, with 6 rats in each group (control, diabetes mellitus, melatonin – diabetes mellitus, and resveratrol + diabetes mellitus). Diabetes was formed by giving streptozotocin to all groups except the control group. Melatonin, 5 mg/kg/day, was given to the melatonin – diabetes mellitus group, and resveratrol, 5 mg/kg/day, was given to the resveratrol + diabetes mellitus group via intraperitoneally for 3 weeks. Interleukin-1 beta, tumor necrosis factor alpha, histone H4, and β-defensin 1 levels were measured in the blood of all rats. The lung, liver, and kidney tissue of all rats were performed as histopathological examinations.

**Results:**

: Whereas there was no difference between the other groups (*P* > .05), interleukin-1 beta levels of the diabetes mellitus group were found to be significantly higher compared with the control group (5.02 ± 2.15 vs. 2.38 ± 0.72 ng/mL; *P* < .05). Whereas histone H4 levels of the diabetes mellitus group were higher compared with the control and resveratrol + diabetes mellitus groups (7.53 ± 3.30 vs. 2.97 ± 1.57 and 3.06 ± 1.57 ng/mL; *P* < .05), the β-defensin 1 levels of the diabetes mellitus group were lower compared with control and resveratrol + diabetes mellitus groups (7.6 ± 2.8 vs. 21.6 ± 5.5 and 18.8 ± 7.4 ng/mL; *P* < .05). β-Defensin 1 levels were moderately inversely correlated with interleukin-1 beta and histone H4 levels (rs > −0.50, *P* < .01). Histopathological changes found in favor of target cell damage in the diabetes mellitus group were not observed in resveratrol + diabetes mellitus group.

**Conclusion:**

Resveratrol may be used as a biotherapeutic agent, which significantly reduces diabetes-induced histone H4 and interleukin-1 beta-mediated liver and other target organ damage.

**Main Points**

- Research on many molecules to be used in the management of diabetes is still ongoing.
- Resveratrol may be used as a therapeutic agent in diabetes with its efficacy on histone H4 and interleukin-1 beta-mediated cell damage.
- Moreover, resveratrol may prevent target organ damage in diabetic cases.

## Introduction

Diabetes mellitus (DM), characterized by chronic hyperglycemia, is a multifaceted endocrine disorder. It causes damage to target organs such as liver, kidney, and cardiovascular system due to vascular and neural pathologies.^1^ Inflammatory and immunological processes play a significant role in the development of all these complications related to DM.^2^ Therapeutic approaches regarding inflammatory and immunological processes of DM are the main concerns about the disease.

Investigating the efficacy of biological agents such as resveratrol and melatonin may provide a very important threshold to be overcome in the management of DM.^2,3^ Interleukin-1 beta (IL-1β), interferon gamma (IFN-γ) resveratrol and melatonin may provide a very important threshold to be overflammation of the pancreas and induce the release of beta-defensins. Moreover, their monitoring in decision-making processes is crucial in the management of DM.^2,3^ On the other hand, it is enlightening to investigate epigenetic reflections of diabetic inflammation using new markers such as histone H4 (H4). This investigation also helps to assess the biotherapeutic efficacy of antidiabetic agents.^3,4^

Research on many molecules to be used in the control of DM is still ongoing. Among them, resveratrol, a herbal product, was found to be remarkable.^2-4^ However, there is no definite conclusion about the use of resveratrol as an antidiabetic agent. Moreover, there is not enough evidence that resveratrol has preventive effects on DM-related target organ damage. In our study using a rat model, it was aimed to investigate the effects of resveratrol in comparison with melatonin on tumor necrosis factor alpha (TNF-α), IL-1β, H4, and beta-defensin 1 mediated anti-inflammatory, immunogenic, and histopathological alterations on target organs of diabetic rats.

## Materials and Methods

### Experimental Method

In the study, 24 Sprague–Dawley–Albino male rats (16-20 weeks old) with an average weight of 297 ± 20 g were used. The rats were kept in a 12-hour light–dark cycle, with the moisture of approximately 55%-60%, and ventilated room with a temperature of 20-22°C where the floors of special cages were cleaned daily. Rats were fed with standard pellet chow and tap water.

Rats were randomly categorized into 4 groups, with 6 rats in each group. Streptozocin (STZ) (HPLC based on ≥75% α-anomer and purity of ≥98%, Sigma-Aldrich, Saint Louis, Mo, USA) was used to induce experimental diabetes in groups of rats fasted for 12 hours, excluding group C. 0.5 mL of STZ (60 mg/kg) dissolved in the buffer of Na citrate (0.1 M, pH 4.5) was applied as a single dose intraperitoneally (IP). Glucose measurement was made within a glucometer (IME-DC, Germany) device using a blood drop collected from the dorsal vein of the foot of all rats via a thin lancet. It was accepted that diabetes developed with a blood glucose level of 200 mg/dL and above in rats. In addition, all rats in the control group had blood glucose levels below 135 mg/dL.^5^

### Melatonin and Resveratrol Administration Protocol

Group I (control group: C): A single dose of sodium-citrate buffer was applied to healthy rats, in order to eliminate metabolic effects and interferences due to solvents. In addition, a 25% ethanol-saline solution (ESS) that was used as the solvent of resveratrol and melatonin was applied via IP every day for 3 weeks in the same amount as the other groups.

Group II (diabetes group: DM): Only 25% ESS was given as IP every day for 3 weeks in this diabetic group.

Group III (DM + melatonin group: DM-M): Melatonin solution prepared by dissolving in a 25% ESS (5 mg/kg/day) was applied to this group as IP for 3 weeks in this diabetic group. Immediately after the application, the rats were taken to the dark environment.^6^

Group IV (DM + resveratrol group: DM-R): Resveratrol solution prepared by dissolving in a 25% ESS (5 mg/kg/day) was applied to this group as IP for 3 weeks in this diabetic group.

In a study in a rat model of peripheral neuropathy, pretreatment with orally administered melatonin (5/10/50 mg/kg/day) was protective, and all 3 doses revealed limited the development of mechanical hypersensitivity.^7^ Considering that the bioavailability of IP administration would be higher, the dose of melatonin was determined as 5 mg/kg/day for this study. In addition, in a study in which a diabetic rat model with cerebral ischemia was created, a significant decrease in the percentage of cerebral infarction with a single dose of 5 mg/kg resveratrol was the determinant of the resveratrol dose in this study.^8^

Resveratrol (≥99% purity, HPLC) and melatonin (≥98% purity, TLC) from Sigma-Aldrich (Saint Louis, Mo, USA) were used in this current experimental study.

### Surgical Procedure and Blood Sample Collection

The same surgical procedure and anesthesia protocol were applied to all rats. A solution consisting of a mixture of xylazine (10 mg/kg) and ketamine (100 mg/kg) was administered as an anesthetic agent approximately 30 minutes before the surgical operation. Anesthesia was maintained using one-third of the initial dose.

At the end of the application procedure in the experimental study, intracardiac blood was collected following the surgical procedure under anesthesia. After blood samples were taken, sacrification was applied to all rats by cervical dislocation procedure. Subsequently, liver, kidney, and lung tissue samples of rats were taken into special containers containing formalin for histopathological analysis.

The blood collected into serum tubes from all rats was centrifuged at 3500 rpm for 10 minutes. The sera obtained from blood were stored in a deep freezer at −80°C until the analysis day. Tumor necrosis factor-α, H4, IL-1β, and β-defensin 1 levels were measured in these sera brought to room temperature on analysis day.

### Biochemical Analyses

Rat IL-1β, H4, β-defensin 1, and TNF-α levels: Rat IL-1β, H4, β-defensin 1, and TNF-α levels were measured in an ELISA device using the sandwich ELISA method (Elabscience Biotechnology, Wuhan, China). The sensitivity of the rat IL-1β, H4, β-defensin 1, and TNF-α assays was reported as 0.08 ng/mL, 0.25 ng/mL, 0.23 ng/mL, and 9.38 pg/mL, respectively. Moreover, the intra- and inter-assay CVs of all 4 were reported as <10%**. **

All ELISA measurements were made using the BioTek Epoch 2 Microplate ELISA Reader (USA).

### Histopathological Analysis

Liver, kidney, and lung tissue samples from sacrificed rats were fixed using formalin (10%), then dehydrated and covered with paraffin. Sections of 5 μm thickness were taken from tissue blocks and stained with hematoxylin–eosin for histopathological evaluation. Modified semiquantitative scores were used for the evaluation of histopathological changes; [0, none; 1, mild; 2, moderate; 3, severe grade].^9^ Samples were evaluated and visualized with imaging-assisted binocular light microscopy (Olympus BX50, Japan).

### In Silico Gene Target Prediction

In this study, a gene function indicator method called STRING was used to show the relationships between genes and protein datasets through an interactive functional association network.^10^ Protein–Protein Interaction (PPI) Networks of cooperating H4 and β-defensin related genes were determined using the STRING method. 

### Ethical Committee Approval

Ethical permissions for the study were obtained from the University of Health Sciences, Hamidiye Animal Experiments Ethics Committee, Turkey (approval no: 2019-08, Date: 16.10.2019).

### Statistical Analysis

Statistical Package for the Social Sciences software (version 20, IBM Corp., Armonk, NY, USA) was used for the statistical evaluation of the research data. The Kruskal–Wallis (nonparametric analysis of variance) test was applied for the analysis of data with more than 2 independent groups and no Gaussian distribution. Dunn’s Multiple Comparison Test was performed for intergroup comparisons, when the *P* value was found to be statistically significant. Spearman Rank Correlation (rs) analysis was used in the correlational evaluations of the data with less than 30 samples.

## Results

### Characteristics of Rats

There was no significant statistical difference between experimental groups in terms of rats’ age and weight (*P* > .05) (Table 1).

### Biochemical Results

**Figure 1.**
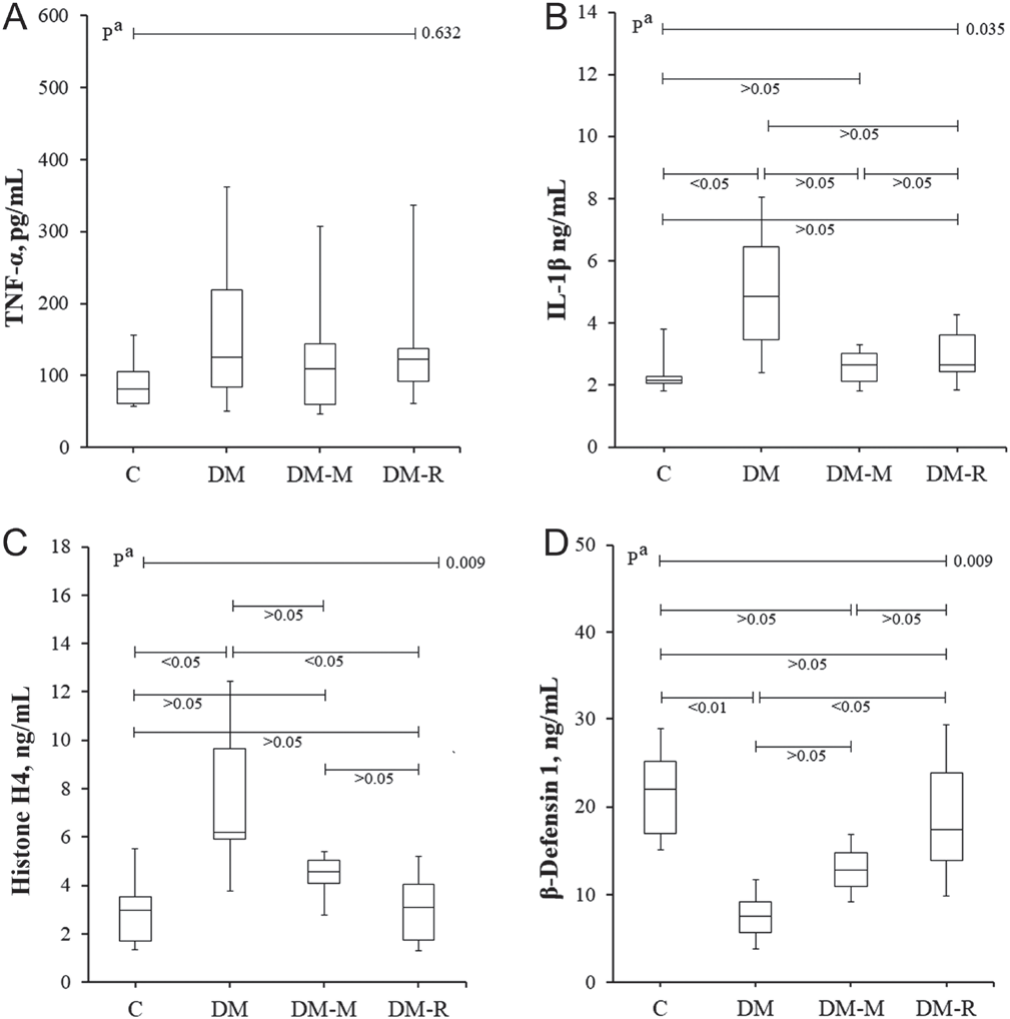
Comparison of (A) TNF-α, (B) IL-1β, (C) H4, and (D) β-defensin 1 levels among control, DM, DM-M, and DM-R groups. The Kruskal–Wallis test (nonparametric ANOVA) was used for all statistical comparisons. No significant difference was observed between the groups in terms of TNF-α. It was observed that IL-1β levels increased significantly in the C group and decreased almost to the control levels in the DM-M and DM-R treatment groups. Compared to the DM group, histone H4 levels were significantly decreased in the DM-R group given only resveratrol, while β-defensin 1 levels were significantly increased. ANOVA, analysis of variance; C, control group; DM, diabetes mellitus group; DM-M, DM + melatonin group, DM-R, DM + resveratrol group; IL-1β, Interleukin-1 beta; TNF-α, tumor necrosis factor alpha.

Although the DM group had higher TNF-α levels compared with the other groups, the difference was not statistically significant (*P* > .05) (Table 2 and Figure 1A). The DM group’s IL-1β levels were significantly higher compared with those of the C group (*P* < .05). In DM-M and DM-R groups, IL-1β levels were found to decrease almost to the levels of C group (Figure 1B).

Whereas the H4 levels of the experimental groups were compared, the H4 levels in the DM group were higher than those of the C and DM-R groups, and the differences were statistically significant (*P* < .05) (Figure 1C). No difference was found between DM and DM-M groups in terms of β-defensin 1 levels (*P* > .05). The DM group had significantly lower levels of β-defensin 1 than the C and DM-R groups (*P* < .05) (Figure 1D). It was also found that the DM-R group’s β-defensin 1 levels decreased to the levels of the C group. In terms of H4 and β-defensin 1, the effects in favor of resveratrol compared to melatonin were remarkable.

**Figure 2.**
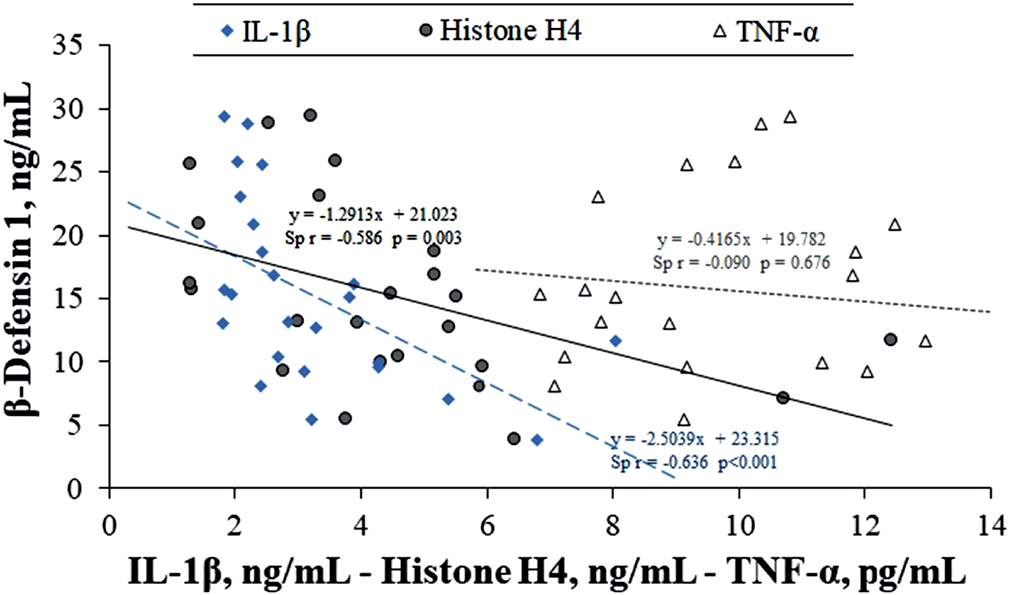
Correlation graph showing the relationship between β-defensin 1 levels and IL-1β, histone H4 and TNF-α levels. The graph shows a moderately significant inverse correlation of β-defensin 1 levels with IL-1β and H4 levels. IL-1β, Interleukin-1 beta; TNF-α, tumor necrosis factor alpha.

According to rs analysis results, β-defensin 1 levels were a moderate negative relationship with IL-1 β and histone H4 levels (rs = −0.636, *P* < .001 and rs = −0.586, *P* = .003, respectively) (Figure 2). There was no statistically significant correlation between β-defensin 1 and TNF-α levels (*P* > .05). Furthermore, a moderately proportional correlation was determined between IL-1β levels and TNF-α and H4 levels (rs = 0.555, *P* = .005, and rs = 0.534, *P* = .007, respectively).

### Histopathological Results

**Figure 3.**
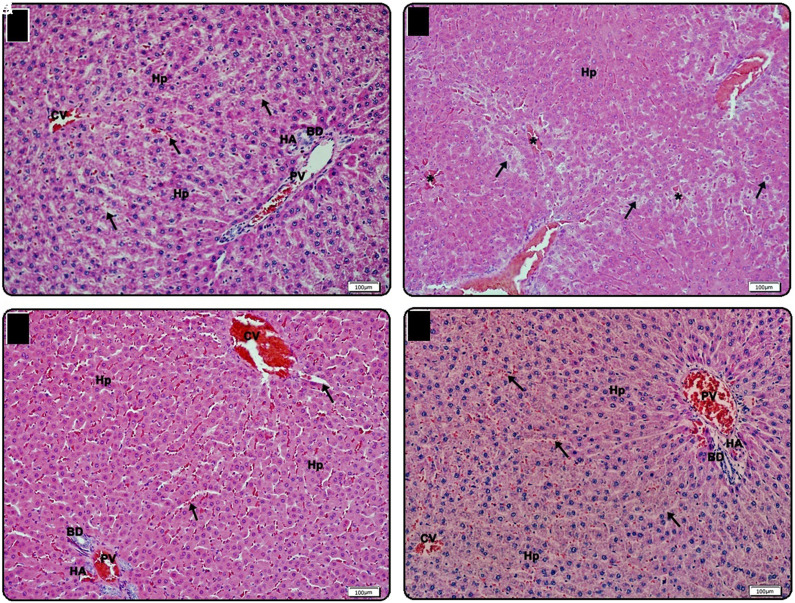
Light microscopic view of liver tissue samples. (A) Belongs to the control group. Hepatocyte (Hp) cords around the CV, and PV, HA and BD, which form the portal triad with sinusoids (arrow) between them, are observed in a normal structure, ×20 (bar: 100 μm). (B) In the DM group, some hepatocytes have balloon degeneration (arrow) and occasional sinusoidal dilatation (*) between hepatocyte (Hp) cords, ×20 (bar: 100 μm). (C) In the DM-M group, hepatocyte (Hp) cords around the CV and occasionally dilated sinusoids (arrow) and PV, HA, and BD forming the portal triad are observed, ×20 (bar: 100 μm). (D) Light microscopic view of liver tissue samples belonging to the DM-R group. CV, hepatocyte cords (Hp), sinusoids (arrow), and PV, HA and BD forming the portal triad are observed in normal appearance, ×20 (bar: 100 μm). BD, bile duct; CV, central vein; DM, diabetes mellitus group; DM-M, DM + melatonin group, DM-R, DM + resveratrol group; HA, hepatic artery; IL-1β, Interleukin-1 beta; PV, portal vein.

In the light microscopic examination of the liver tissue samples of group C, the central vein, hepatocyte cords, and portal triad structures forming the classical liver lobule were found to be normal. Sinusoids between cell cords formed by hepatocytes characterized by vesicular nuclei and Kupffer cells located in this sinusoidal space were intact. It was observed that the triad consisting of the vena porta, hepatic artery, and bile duct, known as the portal triad, was normal (Figure 3A). In the DM group, balloon degeneration and sinusoidal dilatation were noticed in hepatocytes. However, in general, the central vein and portal triad structures remained intact (Figure 3B). Moreover, balloon degenerations were not observed in both treatment groups, but sinusoidal dilatation continued in some areas in the DM-M group (Figure 3C and 3D). Semiquantitative degeneration severity scoring of liver tissues is summarized in Table 3.

**Figure 4.**
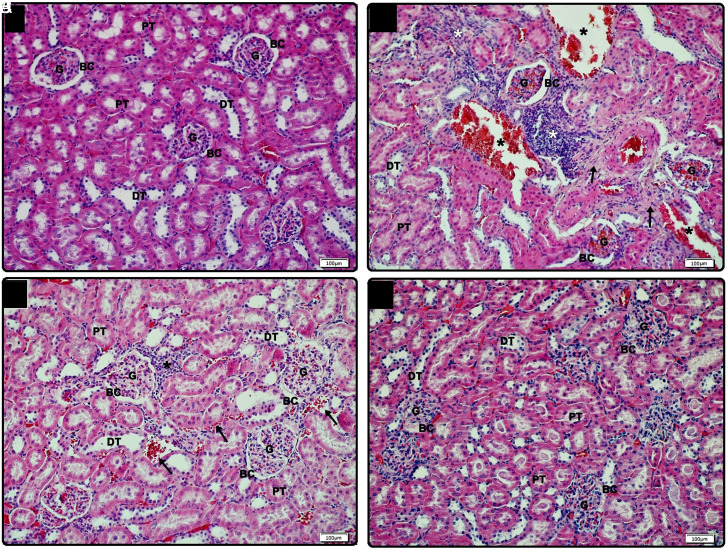
Light microscopic view of kidney tissue samples. (A) In the control group, normal glomerulus (G), BC, PT, and DT are observed, ×20 (Bar: 100 μm). (B) In the DM group, glomerular congestion in the cortex, enlargement of the BC space with hemorrhagic areas (black*), lymphocyte infiltration (white*), and fibrotic areas (arrow) are observed between the tubules. In the DM group, glomerular shrinkage, glomerular vacuolization (arrow) and enlargement of Bowman’s capsular space (BC) are observed in the cortex, ×20 (nar: 100 μm). (C) In the DM-M group, while the glomerulus (G), BC, PT, and DT are in normal structure in the cortex, hemorrhage (arrow) and lymphocyte infiltration (*) are observed in the intertubular area, ×20 (bar: 100 μm). (D) Normal glomerulus (G), BC, PT, and DT are observed in the DM-M group, ×20 (bar: 100 μm). BC, Bowman’s capsule; DT, distal tubule; DM, diabetes mellitus group; DM-M, DM + melatonin group, DM-R, DM + resveratrol group; H&E, hematoxylin–eosin; PT, proximal tubule.

In the light microscopic examination of kidney tissue samples of rats in group C, cortex and medulla layers were in normal structure. The renal body consisting of Bowman’s capsule and glomerular capillary network in the cortex, and the tubule system consisting of proximal tubule, distal tubule, and loop of Henle are intact (Figure 4A). In the DM group, localized lymphocyte infiltration was observed in the cortical region. While glomerular congestion was observed in some renal bodies, glomerular vacuolization was observed in some. In addition, enlargement of the capsular space of the Bowman was determined due to glomerular shrinkage. Hemorrhagic areas and fibrosis were detected between the tubules (Figure 4B). While degenerative changes in the renal body, tubule system, and intertubular areas were observed to decrease significantly in the DM-R group, it was noted that lymphocyte infiltration and intertubular hemorrhage in the cortical region continued in the DM-M group (Figure 4C and 4D). Semiquantitative degeneration severity scoring of renal tissues is summarized in Table 4.

**Figure 5.**
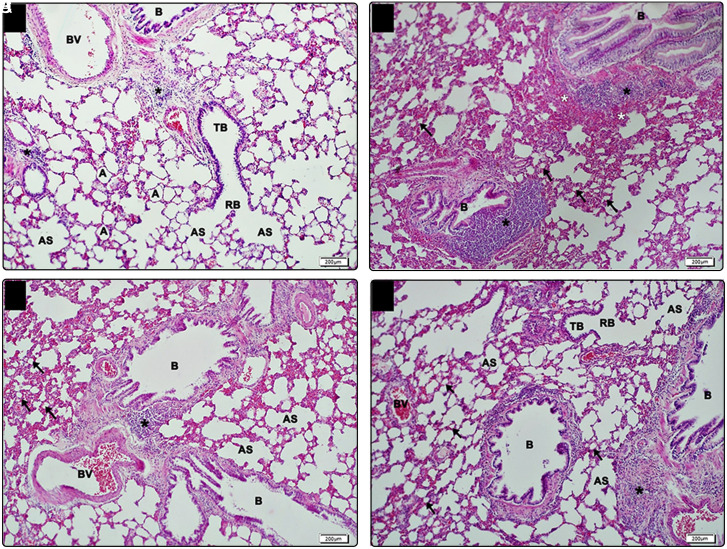
Light microscopic view of lung tissue samples. (A) In the control group, respiratory part appears normal in the lung parenchyma at bronchiole (B), TB, RB, AS, and alveolar (A) levels, ×10 (bar: 200 μm). (B) In the DM group, some lymphoid tissue (black*) increase is observed in the bronchioles (B) with normal appearance. There is a narrowing in the diameter of the alveolar (A) together with the hemorrhagic areas (white*) in places, ×10 (bar: 200 μm). (C) Light microscopic view of lung tissue samples belonging to the DM-M group. While the respiratory part is relatively normal at the levels of bronchioles (B), TB, RB, and AS in the lung parenchyma, it is observed that narrowing in the diameters of some alveoli (arrows) continues. Lymphoid tissue (*), BV, ×10 (bar: 200 μm). (D) In the DM-R group, bronchioles (B), TB, RB, and AS are in normal structure in the lung parenchyma, and narrowing in the diameter of some alveoli (arrow) is observed. Lymphoid tissue (*), BV, ×10 (bar: 200 μm). A, alveolar; AS, alveolar sac; B, bronchiole; BV, blood vessel; DM, diabetes mellitus group; DM-M, DM + melatonin group, DM-R, DM + resveratrol group; H&E, hematoxylin–eosin; RB, respiratory bronchiole; TB, terminal bronchiole.

In the light microscopic examination of the lung tissue samples of rats in group C, the bronchiole, alveolar duct, alveolar sac, and alveolar structures were normal. Large bronchioles with single-layered ciliated columnar epithelium, smooth muscle, and lymphoid tissue were followed by terminal bronchioles and respiratory bronchioles characterized by single-layered cuboidal epithelium. Respiratory bronchioles were opening into alveolar sacs through alveolar ducts (Figure 5A). In the DM group, although the respiratory part of the lung at the bronchiole level was normal, a slight increase in the lymphoid tissue close to the bronchioles and hemorrhagic areas was observed. At the alveolar level, there was a significant reduction in alveolar diameter (Figure 5B). The difference between DM-M and DM-R groups was not statistically significant. While wide bronchioles and terminal and respiratory bronchioles showed normal histological structure in both groups, a slight decrease in lymphoid tissue contents was detected compared to the diabetes group (Figure 5C and 5D). Semiquantitative degeneration severity scoring of lung tissues is summarized in Table 5.

### In Silico Analysis Results

**Figure 6.**
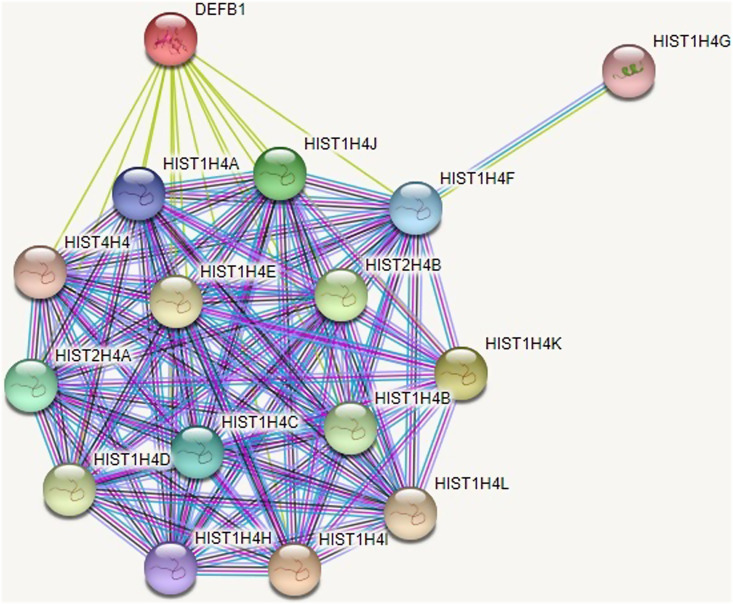
Protein–protein interaction network of multiple genes that H4 is encoded and relationship between DEFB1 gene. Pink, experimentally determined (known interactions); blue, from curated databases (known interactions); yellow, text-mining; green, gene neighborhood (predicted interactions); black, coexpression. The interaction score was set to high confidence (0.992).

Through functional enrichments in the PPI network, the biological process, molecular function, and cellular component of these genes revealed that they are significantly engaged in telomere maintenance, DNA, RNA, and protein binding, and nucleosome mechanism (Figure 6).

## Discussion

According to the literature, this is the first study to evaluate the relationships between resveratrol, melatonin, H4, and β-defensin 1 in an experimental rat diabetes model and to examine differences in end-organ damage.

In case of DM, micro- and macrovascular complications, life-threatening problems, and target organ damage such as liver and kidney occur.^11,12^ Therefore, it is very important to use appropriate experimental modules in order to achieve therapeutic targets for DM. For this purpose, single-dose STZ is one of the most commonly used agents to induce DM in rats.^13,14^

Despite advances in DM treatment, adequate diabetic control cannot be achieved in the majority of patients.^11,12^ Therefore, new treatment options and additional applications are needed to achieve glycemic control. For this purpose, it has been suggested that resveratrol, a natural phytopharmaceutical product with cardioprotective and neuroprotective, antidiabetic, antioxidant, anti-inflammatory, and anticarcinogenic properties, can be used.^15,16^ It has also been suggested that melatonin, which is a highly potent antioxidant secreted naturally from the pineal gland, may reduce the complications of DM^16^ and hepatocellular damage by increasing the production of insulin growth factor and stimulating insulin receptor tyrosine phosphorylation.^17^ In this current study, resveratrol’s lowering efficacy of IL-1β levels confirms the suggestions above and explains the anti-inflammatory mechanism. In addition to these, IL-1β is also involved in several critical cellular activities such as proliferation, activation, and differentiation, as well as induces leukocyte phagocytosis and chemotaxis.^18-20^ In this current study, we expected a significant decrease in TNF-α levels, a proinflammatory cytokine such as IL-1β, in treatment groups. However, the absence of a decrease was probably attributed to the fact that TNF is a locally released cytokine, and therefore, the increase and decrease in tissues cannot be adequately reflected in the systemic circulation.^21^ It should be remembered that intracardiac blood was taken for TNF-α measurement in this study. In addition, the small sample size may be the reason why the difference between the groups was not statistically significant.

Histones and their posttranslational modifications (such as acetylation, methylation, and phosphorylation) play critical roles in gene transcription, a reflection of the cellular response. Histones can cause systemic inflammatory and toxic responses by activating inflammatory pathways when they are released out of the cell, and they act as damage-associated model molecules. The occurrence and progression of many autoimmune, inflammatory, and malignant diseases are associated with extracellular histone levels. Therefore, it is thought that blood histone levels can be used as new therapeutic targets.^22^ By reason H4 levels were determined to be significantly higher in the DM group in this current study, it can be suggested that diabetes has a chronic inflammatory process compatible with the given information above. In addition, the linear relationship we found between H4 and IL-1β levels by correlation analyses and positive findings in the histopathological findings of liver and kidney tissue in the resveratrol group also support this interpretation. In the early stages of DM, nitric oxide (NO) synthesis decreases with an increase in the production of free oxygen radicals (ROS). Vascular dilatation and inflammation occur as a result of this oxidative stress and endothelial dysfunction.^22^ Consistent with previous literature, sinusoidal dilatation, hemorrhagic areas, and lymphocyte infiltration due to loss of endothelial cell function were observed in both liver and kidney tissue sections in our study. We found that especially resveratrol treatment showed positive effects on these histopathological changes. This finding is consistent with the knowledge that resveratrol protects the liver from hepatic damage caused by ROS and inflammatory cytokines.^23^ As is known, mitochondria are the main production source of ROS. Excessive mitochondrial fragmentation induces T-cell toxicity by causing ROS accumulation.^24-26^ Thus, improvement of mitochondrial dynamics may contribute to the biotherapeutic effect of resveratrol. Similar to the above data, consistent with our current findings, the protective effect of resveratrol toward oxidative stress and inflammation in the kidney was reported previously.^27^ However, since oxidative stress markers were not studied in this study, the relationship between our current findings and oxidative stress could not be tested. Based on these findings, we think that resveratrol can significantly reduce histone-mediated target organ damage due to diabetes.

The α- and β-defensins, which were defined as antimicrobial peptides of the innate immune system in the past, are now seen as alarm signals with important roles in inflammation and innate immunity.^28^ β-defensins play an important role in immunological reactions against viruses and bacteria and in the formation of inflammatory responses. In addition to being widely expressed by skin and gastrointestinal, urogenital, and respiratory epithelial cells, they are also expressed by leukocytes (especially β-defensin 1).^29^ Németh et al^30^ suggested that α and β-defensins play a critical role in diabetes development and its chronic addition, the decrease in β-defensins was shown as one of the possible causes of the frequent infections in diabetic patients. Consistent with the information above, in this current study, the fact that serum β-defensin 1 levels were lower in the DM group than in the C group was interpreted as a sign of decreased protective efficacy of β-defensins and therefore an increase in inflammation. In addition, the negative correlation of β-defensin 1 levels with serum IL-1β and H4 levels in this current study explains the close relationship of β-defensin 1 with immunological and inflammatory processes. The regression of diabetes-related complications in the liver and other target organ tissues in the resveratrol treatment group also supports this result.

In conclusion, resveratrol can be used as an important biotherapeutic agent that significantly reduces histone and IL-1β-mediated cell damage due to diabetes, supports anti-inflammatory and innate host defense via β-defensin 1, and reduces target organ damage in organs such as liver and kidney.

## Figures and Tables

**Table 1.** Age and Weight of Rats According to Study Groups

**n**	C	DM	DM-M	DM-R	*P*
	6	6	6	6	–
**Age, week**3	18.2 ± 1.2 18.0 (17.0-20.0)	18.3 ± 1.2 18.5 (17.0-20.0)	18.0 ± 1.7 17.5 (16.0-20.0)	18.0 ± 0.9 18.0 (17.0-19.0)	.9557^a^3
**Weight, g**3	302 ± 19 303 (280-332)	298 ± 18 296 (275-320)	293 ± 24 287 (270-339)	296 ± 24 287 (278-342)	.7024^a^3

^a^Kruskal–Wallis test (nonparametric ANOVA): The *P* value is approximate (from chi-square distribution). C, control group; DM, diabetes mellitus group; DM-M, DM + melatonin group, DM-R, DM + resveratrol group.

**Table 2.** Comparison of Serum IL-1β, β-Defensin 1, Histone H4, and TNF-α Levels of the Groups

n	C	DM	DM-M	DM-R	*P*
	6	6	6	6	–
IL-1β, ng/mL	2.38 ± 0.72 2.14 (1.83-3.81)	5.02 ± 2.15 4.84 (2.40-8.03)	2.57 ± 0.60 2.64 (1.80-3.30)	2.95 ± 0.94 2.64 (1.84-4.29)	.0352^a^3
	^*^<0.05, >0.05, >0.05, >0.05, >0.05, >0.05				
Histone H4, ng/mL	2.97 ± 1.57 2.96 (1.34-5.52)	7.53 ± 3.30 6.19 (3.78-12.42)	4.40 ± 0.95 4.55 (2.77-5.41)	3.06 ± 1.57 3.11 (1.31-5.19)	.0085^a^3
	^*^ <0.05, >0.05, >0.05, >0.05, <0.05, >0.05				
β-Defensin 1, ng/mL	21.6 ± 5.5 22.0 (15.1-28.8)	7.6 ± 2.8 7.6 (3.8-11.7)	12.9 ± 2.9 12.9 (9.3-16.9)	18.8 ± 7.4 17.4 (9.9-29.3)	.0018^a^3
	^*^<0.001, >0.05, >0.05, >0.05, <0.05, >0.05				
TNF-α, pg/mL	91 ± 38 82 (57-156)	164 ± 119 126 (50-362)	128 ± 97 109 (47-307)	145 ± 99 122 (61-337)	.6320^a^3

^a^Kruskal–Wallis test.

^*^If *P* value is <.05, *P* values of between groups (respectively, C and DM, C and DM-M, C and DM-R, DM and DM-M, C and DM-R, and DM-M and DM-R) are compared with posttest.

C, control group; DM, diabetes mellitus group; DM-M, DM + melatonin group; DM-R, DM + resveratrol group; IL-1β, interleukin-1 beta; TNF-α, tumor necrosis factor alpha.

**Table 3.** Semiquantitative Histopathological Scoring of Liver Tissues

Histopathologic Degenerations	C	DM	DM-M	DM-R
Balloon degeneration	−	**+++**3	−	−
Sinusoidal dilatation	−	**+++**3	−	**+**3
Portal triad degeneration	−	−	−	−
Lymphocyte infiltration	−	−	−	−
Hemorrhage	−	**+++**3	−	−
Fibrosis	−	−	−	−

Scoring was done as follows: none (−), mild (+), moderate (++), and severe (+++).

C, control; DM, diabetes mellitus; DM-M, DM + melatonin; DM-R, DM + resveratrol.

**Table 4.** Semiquantitative Histopathological Scoring of Renal Tissues

Histopathologic Degenerations	C	DM	DM-M	DM-R
Glomerular vacuolization	−	+++	−	−
Glomerular congestion	−	+++	−	−
Enlargement of capsular space in BC	−	+++	−	−
Lymphocyte infiltration	−	+++	−	+
Hemorrhage	−	+++	−	+
Fibrosis	−	+++	−	−

Scoring was done as follows: none (−), mild (+), moderate (++), and severe (+++).

BC, Bowman’s capsule; C, control; DM, diabetes mellitus; DM-M, DM + melatonin; DM-R, DM + resveratrol.

**Table 5.** Semiquantitative Histopathological Scoring of Lung Tissues

Histopathologic Degenerations	C	DM	DM-M	DM-R
Bronchiolitis	**+**3	**+++**3	**+**3	**++**3
Alveolit	−	−	−	−
Shrinking of alveoli	−	**+++**3	**++**3	**++**3
Hemorrhage	−	**+++**3	**++**3	**++**3
Fibrosis	−	−	−	−
Edema	−	−	−	−

Scoring was done as follows: none (−), mild (+), moderate (++), and severe (+++).

C, control; DM, diabetes mellitus; DM-M, DM + melatonin; DM-R, DM + resveratrol.
